# Anti‐IL‐5 Vaccination Dampens Allergen‐Specific IgE Levels and Modulates IL‐4 and IL‐5 Th2 Cytokines in Skin Allergy of Mice and Horses

**DOI:** 10.1111/all.70020

**Published:** 2025-08-21

**Authors:** Fadi Jebbawi, Florian Olomski, Victoria Inversini, Giulia Keller, Tanya Rhiner, Nina Waldern, Juwela Lam, Stanislav Pantelyushin, Fabia Canonica, Katharina Birkmann, Pål Johansen, Thomas M. Kündig, Antonia Fettelschoss‐Gabriel

**Affiliations:** ^1^ Department of Dermatology University Hospital Zurich Schlieren Switzerland; ^2^ Faculty of Medicine University of Zurich Zürich Switzerland; ^3^ Evax AG Guntershausen Switzerland; ^4^ Equine Department, Veterinary Faculty Ludwig Maximilians University Munich LMU Oberschleißheim Germany

**Keywords:** allergy, equine, IgE allergen‐specific, IL‐4, IL‐5, insect bite hypersensitivity, vaccination

## Abstract

**Background:**

Skin allergies are among the most frequent types of allergies, where continuous investigation of the pathological immune mechanisms is required for a better understanding and a more effective treatment of the disease. In this study, we aimed to investigate the effect of interleukin (IL)‐5 vaccination on allergen‐specific IgE antibodies as well as T cell cytokine modulation in skin allergy using a mouse model and a naturally occurring disease in horses.

**Methods:**

Ovalbumin (OVA)‐sensitized mice, as well as horses affected by equine insect bite hypersensitivity (IBH) were administered an anti‐IL‐5 vaccination, and allergen‐specific IgE and IgG were quantified in the blood. Additionally, mRNA and protein expression of T cell cytokines of in vitro allergen re‐stimulated murine splenocytes and equine peripheral blood mononuclear cells (PBMCs), as well as in IBH lesional skin biopsies, were investigated using qPCR and ELISA. Clinical signs were recorded by ear swelling in mice.

**Results:**

Our data showed a significant decrease in allergen‐specific immunoglobulins (Igs) in IL‐5‐vaccinated mice, as well as a reduction in allergen‐specific IgE in horses. Furthermore, protein production of T cell cytokines IL‐4 and IFNγ in mice, as well as mRNA expression of IL‐4, IL‐5, IL‐13, and IFNγ in lesional skin of the horses, was significantly decreased upon vaccination when compared to the placebo group. Furthermore, we demonstrated that CD4 ^+^ cells in IBH‐affected horses are highly enriched with the GATA3 transcription factor, responsible for IL‐5 mRNA production and differentiation of Th2 cells. Additionally, increased IL‐4 mRNA expression in IBH horses was shown to be CD4 ^−^ MHC‐II ^−^cell dependent.

**Conclusion:**

IL‐5 vaccination significantly decreased allergen‐specific IgE in both the murine skin allergy model and horses with naturally occurring allergic skin disease, as well as alleviated clinical signs of the diseases. We suggest that the IL‐5 depletion may modulate the IL‐4 levels originating from non‐T cell sources. This is the first study showing that an IL‐5 vaccination leads to a decrease in allergen‐specific IgE levels, potentially suggesting its use in prophylactic settings for high‐risk patients.

## Graphical abstract

This study investigates the effect of IL‐5 vaccination on allergen‐specific IgE levels, as well as mRNA and protein expression of Th1/Th2‐associated cytokines using a murine OVA skin allergy model and the naturally occurring skin allergy IBH in horses. IL‐5‐vaccination significantly decreases allergen‐specific IgE levels, together with Th1/Th2 cytokines such as IL‐4 and IFN‐γ. Targeting IL‐5 with vaccination proves to be both effective and safe over the long term and shows a big potential to be used for a prophylactic effect on exported horses to Culicoides‐infested areas. IBH, Insect bite hypersensitivity; IFN‐γ, interferon gamma; Ig, immunoglobulin; IL‐interleukin; OVA, ovalbumin; Th, T helper.

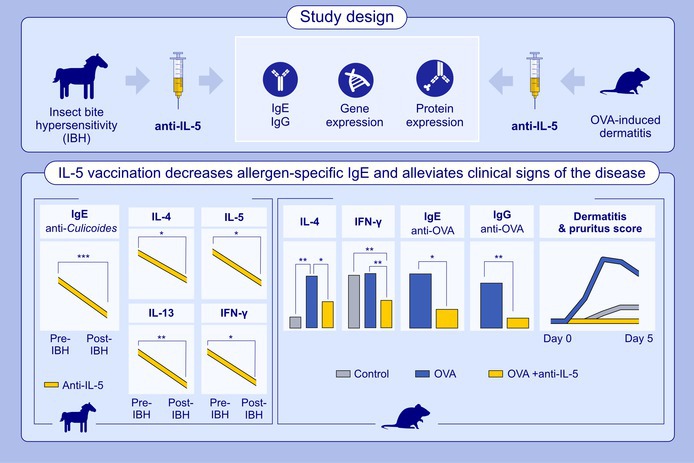

## 1 Introduction

Interleukin (IL‐) 5 is a T helper cell type 2 (Th2) cytokine, known for its major role in various conditions. The latter plays a role not only in allergic diseases such as asthma and atopic diseases, but also in various infections and other inflammatory diseases. Mitson‐Salazar and Prussin reviewed [1] studies in humans and mice, describing the existence of two subpopulations of Th2 cells: IL‐5^+^ pathogenic effector Th2 cells (peTh2) [2] and IL‐5^−^ conventional Th2 cells (cTh2) [3]. In addition, Prussin et al. demonstrated that these two Th2 cell subpopulations could shape the immune response towards an IgE‐mediated or eosinophil‐dominant immunopathology. The role of IgE in horses, including its occurrence in both health and disease such as insect bite hypersensitivity (IBH) has been reviewed in [4, 5]. For instance, in peanut allergy leading to anaphylaxis, cTh2 cells are predominant, whereas peTh2 cells are more prevalent in allergic eosinophilic gastroenteritis [3]. This distinction underscores how the different Th2 subpopulations, specifically peTh2 and cTh2 cells, influence the nature of allergic reactions. PeTh2 cells, with their strong IL‐5 production, play a critical role in mediating eosinophilic responses and are prominent in Type IVb allergic reactions. Conversely, cTh2 cells are key in driving IgE production and are significant in Type I allergic reactions [6, 7]. Understanding these roles is crucial for exploring how Type I and Type IVb allergic reactions are shaped. It is well established that IL‐5 is the master regulator of eosinophil development [8, 9], recruitment, and activation [10]. Thus, targeting IL‐5 is an attractive option to treat eosinophilic diseases. Monoclonal antibodies target either IL‐5 directly (mepolizumab [11, 12, 13] and reslizumab [14, 15]), or the IL‐5 receptor (benralizumab [16, 17, 18]). Following this approach, our group developed a virus‐like particle (VLP)‐based therapeutic vaccine inducing IL‐5 autoantibodies (equine (e)IL‐5 coupled to cucumber mosaic virus‐like particles (CuMV) including a universal epitope of tetanus toxoid (TT), eIL‐5‐CuMVTT for treating equine hypereosinophilic diseases such as IBH). The vaccine proved to be successful, not only in reducing clinical signs of IBH [19, 20] and eosinophilia [19], but also in shifting the eosinophilic subtype profile of the remaining cells towards the subtype associated with healthy horses [21] and providing long‐term reduction of basophil counts [22]. The eIL‐5‐CuMVTT vaccine offers several significant advantages over monoclonal antibodies. These include lower production costs, as well as independence from patient weight, which is an important factor in the treatment of horses. Furthermore, the efficacy and safety were extensively investigated in long‐term studies with good results [23].

There is a necessity to understand the immune cytokine modulation and potential crosslinks of biological pathways in skin allergies for a better treatment approach. In the present study, we investigated the effect of IL‐5 vaccination on allergen‐specific IgE levels in the serum, as well as mRNA and protein expression of Th1/Th2‐associated cytokines using a murine ovalbumin (OVA) skin allergy model and the naturally occurring skin allergy IBH in horses.

For the first time, we demonstrated that IL‐5 vaccination reduced allergen‐specific IgE levels in allergic mice and IBH‐affected horses and further modulated Th1/Th2 cytokines. This suggests a beneficial additional effect of the IL‐5 vaccination, not only interfering with de‐novo eosinophil production, but also acting broadly on other type‐I mechanisms.

## 2 Materials and Methods

### 2.1 Horses & Clinical Study Design

All study horses were client‐owned and privately held by their owners. All clinical studies have been approved by the respective cantonal veterinary authorities under licenses 28711, 33558, and 34167. All horse owners signed informed consent. Horses were screened during the IBH season before the study commenced. A total of 37 horses were included in the vaccination part, whereof 20 horses received the eIL‐5‐CuMVTT vaccine, whereas 17 horses received placebo (vaccine buffer) in a double‐blind, randomized fashion, ensuring blinding for both owners and all study personnel. In addition to the horses in the double‐blind study, blood was collected in peak IBH season, from May to August, from untreated IBH and healthy control horses. Data are shown for all samples measured without any exclusions.

### 2.2 Mice

BALB/c mice were purchased from Inotivco (Horst, the Netherlands). Animals were housed according to the Federation of European Laboratory Animal Science Association (FELASA) guidelines. The animal studies were performed in compliance with the recommendations of the Swiss guidelines for the Care and Use of Laboratory Animals. The protocol used was approved by the Swiss cantonal veterinary authorities under number 28711.

### 2.3 Vaccine Preparation and Immunization

#### 2.3.1 Horses

The production of the equine IL‐5 vaccine, eIL‐5‐CuMVTT, is described in [19]. The vaccine was administered subcutaneously without adjuvants, as previously described [20].

#### 2.3.2 Mice

The murine version of the IL‐5 vaccine, mIL‐5‐Qβ, used in this study was previously described in [24]. CuMVTT VLP alone was used as a control vaccination. Mice were immunized by subcutaneous injection of 50 μg of either the mIL‐5‐Qβ vaccine or VLP, without the addition of an adjuvant, on Days −21, −7, and 7.

### 2.4 MC903‐Based Model of Allergic Dermatitis Using Model Allergen OVA

On Days −21, −7, and 7, 5–7‐week‐old female BALB/c mice (*n* = 6 per group) were either vaccinated using the mIL‐5‐Qβ (“mIL‐5‐VLP”) vaccine or received vaccination using CuMVTT VLP (“VLP”) as a negative control. Two groups of mice received VLP control vaccination; one group received anti‐IL‐5 vaccination. All mice received an intraperitoneal injection of 1 μg OVA (Grade V; Sigma) in alum (Aluminum Hydroxide Gel Adjuvant; Brenntag Biosector, Denmark) for allergen sensitization on Day 0. In addition to the i.p. injection, mice received an epicutaneous sensitization by 6× tape stripping combined with a topical treatment of 2 nmol of MC903 (Cacipotriol; Tocris Biosciences, cat. 2700) together with 200 μg of OVA on the right ear to induce atopic dermatitis on Days 0, 2, 4, 6, 8, and 10. Topical application of MC903 (calipotriol; a low‐calcemic analog of vitamin D3) has been shown to induce keratinocytic TSLP expression and trigger an AD‐like syndrome [25, 26]. Following systemic and topical OVA or PBS sensitization, mice were topically challenged on Days 17, 18, 19, and 20 on the left ear using 6× tape stripping combined with 200 μg OVA, whereas one of the two VLP‐vaccinated control groups received PBS instead. Dermatitis & Pruritus score and ear thickness were measured on Days 17, 18, 19, 20, and 22 on the left challenged ear in all groups. Ear thickness was measured using a caliper. Experimental scheme shown in Figure S1. Of note, no difference for the negative VLP control group using CuMVTT or Qβ alone is expected. In the context of allergy, Qβ VLP control has been studied in Zou et al. and Schmitz et al. [24, 27], showing the absence of inflammation including a similar allergic profile comparable to PBS control.

### 2.5 Dermatitis & Pruritus Score for Mice

The Dermatitis & Pruritus score was calculated as the sum of points related to symptom severity presenting for parameters crust, blood, and lichenification as follows: no sign, 0 points; mild with less than 50% of skin affected, 1 point; strong with equal or more than 50% affected, 2 points.

### 2.6 Blood Withdrawal

#### 2.6.1 Horses

Blood was collected in peak IBH season, from May to August, from healthy (*n* = 19), IBH‐vaccinated (*n* = 20) and IBH‐placebo (*n* = 17) horses from the jugular vein into tubes provided by IDEXX Diavet (Switzerland) for serum preparation and sterile blood collection for peripheral blood mononuclear cell (PBMC) isolation in NH Sodium Heparin VAGUETTE containers (Greiner Bio‐one).

#### 2.6.2 Mice

Blood samples were collected at baseline, the day of first vaccination, 1 week after the second vaccination, and 2 weeks after the second vaccination (Days −21, 0, 22).

### 2.7 Mouse Splenocyte Re‐Stimulation In Vitro

At the end of the MC903/OVA experiment, mice were euthanized. Spleens were collected on ice, smashed through a cell strainer (0.2 mm) followed by centrifugation at 1500 rpm for 5 min at 4°C. Red blood cells were lysed using RBC lysis buffer (BioLegend, cat. 420301) for 3 min at room temperature (RT). Collected splenocytes were washed and re‐suspended at a concentration of 5 × 10^6^ cells/mL in RPMI medium completed with 10% FBS, 1% PenStrep (Thermo Scientific, cat. 15140122), and 1% Glutamine (Thermo Scientific, cat. A2916801). Splenocytes were distributed into wells of a 96‐well U‐shaped plate at 0.5 × 10^6^ cells/100 mL. OVA protein (Grade V; Sigma) was added to the wells at a concentration of 20 μg/mL, or PBS1x was used as a negative control. Cell culture supernatants were collected from all conditions after 72 h of incubation at 37°C with 5% CO_2_ and 95% humidity.

### 2.8 Equine PBMC Isolation and Stimulation In Vitro

Density gradient centrifugation (Ficoll‐Paque Plus, Cytiva, cat. GE17‐1440‐03) was used to isolate PBMCs from diluted heparinized equine blood in PBS1x (Thermo Fischer Scientific, cat. 10010023), using SEPMATE 50 mL tubes (STEMCELL, cat. 85460) according to the manufacturer's instructions. PBMCs were counted and resuspended in RPMI Glutamax 1640 medium (Thermo Fischer Scientific, cat. 72400021) completed with 10% FBS, 1% PenStrep (Thermo Fischer Scientific, cat. 15140122), 1× Non‐Essential Amino Acids (NEAA) (Thermo Fischer Scientific, cat. 11140050), and 1× sodium pyruvate (Thermo Fischer Scientific, cat. 11360070). The cells were stimulated for 18 h with either whole body extract (WBE) of *Culicoides nubeculosus* (1 μg/mL) (Stock: 1 mg/mL; Stallergenes Greer, cat. XPB69X1A2.5), concanavalin A (1 μg/mL) (ConA, Stock: 1 mg/mL; Sigma‐Aldrich, cat. 11028‐71‐0), or medium alone. After 18 h of stimulation, the cells were collected for RNA extraction.

### 2.9 PBMC Staining for Cytokine Production and Sorting

#### 2.9.1 PBMC Staining

Stimulated PBMC were washed three times with PBS, fixed using Cytofix/Cytoperm (BD Biosciences, cat. 554714) and incubated for 20 min in the dark. Then cells were stained with the following reagents in PBS for 20 min at RT, protected from light: Near‐IR‐viability dye (Thermo Fischer Scientific, cat. L34976), FITC‐CD4 (Biorad, cat. MCA1078F, clone CVS4), PE‐MHC II (Biorad, cat. MCA1085PE, clone CVC20) and AF647‐IFNγ (Biorad, cat. MCA1783A647, clone CC302) (10^5^ cells/30 μL). Following this, the cells were resuspended in 300 μL PBS1x for Fluorescence Activated Cell Sorting (FACS) analysis using BD LSR Fortessa II (BD Biosciences). Data analysis was performed using FlowJo software version 10.6 (BD Biosciences).

#### 2.9.2 CD4^+^ Sorting

Stained stimulated PBMCs (10^6^ cells/200 μL in complete RPMI) were prepared for CD4^+^ cell sorting as shown in the gating strategy (Figure S2) using the BD Aria III sorter (BD Biosciences). Post‐sorting, the cells were assessed for purity, washed with PBS1x, and resuspended in RNA lysis buffer from the Cells‐to‐Ct kit (Thermo Fischer Scientific, cat. A35381) for subsequent qPCR analysis, following the manufacturer's instructions.

### 2.10 Punch Biopsies

Punch biopsies (2 mm) from lesional skin of vaccinated (*n* = 15) and placebo (*n* = 6) horses were collected into RNAlater stabilization solution (Thermo Fischer Scientific, cat. AM7022). Lesional skin biopsies were collected while being blinded for the treatment and showed at least three of the following clinical parameters, that is, alopecia, blood/exudate, scales, crust, lichenification, swelling.

### 2.11 Total RNA Extraction, RT and qPCR

#### 2.11.1 RNA Extraction and Reverse Transcription

RNA was extracted from skin punch biopsies of horses and ear punch biopsies from mice using Trizol (Sigma, cat. T9424). The RNA concentration was quantified using a Nanodrop spectrophotometer. 1000 ng of RNA was treated with DNAse I (Ambion, cat. AM2222) and transcribed into cDNA using PrimeScript RT kit (Takara Bio, cat. RR037A) according to the manufacturer's instructions.

#### 2.11.2 qPCR

Real‐time PCR quantitative mRNA analyses were performed on the Viia7 instrument using Fast Syber Green master mix (Thermo Fischer, cat. 4385612). Data were normalized to the expression levels of the endogenous control genes GAPDH, 18S, and beta Actin. Individual gene expression analysis was performed using the $2^{-∆C_{t}}$ (*C*_t_ gene of interest—*C*_t_ housekeeping gene) method. The housekeeping genes used for normalization were GAPDH for skin punch biopsies and 18S for equine sorted cells after showing no variability between the samples. Primers used for RT‐qPCR are listed in Table S1.

### 2.12 Enzyme‐Linked Immunosorbent Assay (ELISA)

#### 2.12.1 Anti‐OVA IgG and IgE ELISA in Mice

Described in [28].

#### 2.12.2 Anti‐Culicoides IgG and IgE ELISA in Horses

Maxisorp 96‐well ELISA plates (Nunc; Thermo Fisher) were coated overnight at 4°C with *Culicoides* whole‐body extract (WBE) at 10 μg/mL and then blocked with 5% blocking milk solution (Thermo Scientific, Waltham, Mass) for 2 h at room temperature (RT). Serum dilution for IgE was 1:5 and for IgG 1:10 followed by a 1:5 serial dilution. For *Culicoides*‐specific IgG detection, goat anti‐horse IgG conjugated with horseradish peroxidase (HRP) (1:10,000) (Jackson, 108‐035‐008) was used. For *Culicoides*‐specific IgE detection [29, 30, 31, 32], mouse anti‐horse IgE (1:2000) (Bio‐Rad, cat. MCA5982GA), followed by goat anti‐mouse IgG (H + L) HRP (1:2000) was used. The plate was developed using TMB substrate (Thermo Scientific, cat. 34021), and 2M sulfuric acid solution was used to stop the reaction. Absorbance was measured at 450 nm using a Tecan Spark spectrophotometer (Tecan, Grödig, Austria). It is known that horses have significantly higher levels of total IgE compared to SPF mice [33] or humans [4]; therefore, IgG depletion may be less relevant in this context and is typically not done in allergen‐specific IgE quantification in horses [34].

#### 2.12.3 Influenza NP‐ELISA

A commercial diagnostic kit designed to detect antibodies against the internal nucleocapsid of the influenza A virus (ELISA ID Screen Influenza A Antibody Competition Multi‐Species kit, IdVet, Montpellier France, cat. FLUACA) was used in this study; tests were performed according to the manufacturer's instructions. The intensity of color reaction was measured at 450 nm with a spectrophotometer (Tecan, Grödig, Austria). Serum samples of horses were collected between 4 and 8 weeks post the horses' annual influenza vaccination and only when influenza vaccination had been administered after establishment of eIL‐5‐immunity for eIL‐5‐vaccinated horses, that is, from 4 weeks post second vaccination in prime‐boost horses (1st year vaccination) or 4 weeks post a booster (2nd year vaccination). According to the specification of the manufacturer's, the result was expressed as a competition percentage (CP) corresponding to [S/N%] = 100 × (OD sample)/(OD negative control). A sample with CP ≤ 45 was considered positive; a sample with 45 < CP < 50 was considered doubtful; and a sample with CP ≥ 50 was considered negative.

#### 2.12.4 IL‐4 and IFNγ Mouse ELISAs

The OptEIA set for mouse interleukin‐4 (IL‐4) (BD Biosciences, cat. 555232) and the OptEIA set for mouse interferon‐γ (IFNγ) (BD Biosciences, cat. 555138) were used. ELISAs were conducted according to the manufacturer's manual.

### 2.13 Statistics

Data were tested for normal distribution. Data are presented as mean ± standard error of the mean (SEM) when normally distributed or median ± interquartile range (IQR) when it is not. The significance of the differences between the groups were determined either with one‐way ANOVA test followed by Tukey's multiple comparisons test or by Kruskal–Wallis test followed by Dunn's Multiple Comparisons post‐test. Mann–Whitney *U* test was used to compare differences between two groups. Wilcoxon test was used for pair analysis. No outliers were excluded. Statistical significance is indicated for the following *p* values: *p < 0.05; **p < 0.01; ***p < 0.001; ****p < 0.0001. Graphs and statistical analysis were performed using GraphPad Prism software (version 9; GraphPad, La Jolla, CA, USA).

## 3 Results

### 3.1 IL‐5 Vaccination Alleviates Cutaneous Allergic Symptoms in Allergic Mice

**FIGURE 1:**
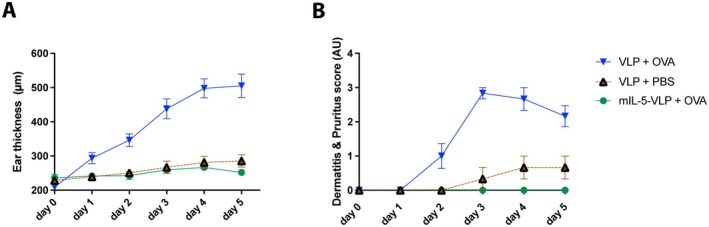
Ear thickness and Dermatitis & Pruritus score in mice during a 5‐day topical OVA challenge. Mice (*n* = 6 per group) are either vaccinated using mIL‐5‐Qβ (mIL‐5‐VLP) or receive control vaccination using the VLP CuMVTT (VLP) and receive topical challenge of ears by OVA or PBS with Group 1, mIL‐5‐VLP + OVA; Group 2, VLP + OVA; Group 3, VLP + PBS. (A) Ear thickness upon challenge (left ear) of measured in μm from Day 0 to Day 5. (B) Dermatitis & Pruritus total score from Day 0 to Day 5. Day 0 corresponds to Day 17 in the experiment plan according to Figure S1.

Mice received three vaccinations either using mIL‐5‐Qβ vaccine (mIL‐5‐VLP) or using a VLP control CuMVTT (VLP) as control. In between the 2nd and 3rd vaccinations, OVA mice were sensitized for OVA with an intraperitoneal injection of OVA/alum and a topical sensitization of the right ear using OVA/MC903 combined with tape stripping. After the 3rd vaccination, OVA‐sensitized mice were challenged with OVA on the left ear, which remained untreated during the sensitization phase. A negative control group received PBS instead. The epidermis of OVA‐challenged mice (VLP + OVA) exhibited a significant increase in ear thickness on Day 1 (40.43%), Day 2 (65.62%), Day 3 (109.4%), Day 4 (138.19%) and Day 5 (141.62%) when compared to Day 0 (Figure 1A) (corresponding to Day 17 of the experimental scheme; Figure S1). In contrast, and as expected, ears of PBS‐challenged control mice (VLP + PBS) did not show meaningful swelling; nevertheless, a slight increase from baseline of 24.85% at Day 5 was found, probably due to repeated measurement of ear thickness. Mice vaccinated with mIL‐5‐Qβ vaccine and OVA‐challenged showed a significant decrease in ear thickness (eIL‐5‐VLP + OVA) when compared to the OVA‐challenged placebo vaccinated group (VLP + OVA). Further, the reduction of the ear swelling upon mIL‐5 vaccination was to a similar extent to the level of the negative control group with PBS challenge (VLP + PBS) (Figure 1A).

The Dermatitis & Pruritus score in OVA‐challenged mice (VLP + OVA) increased with time, from Days 2, 3, 4, and 5 by an average of 1, 2.5, 2, and 1.5 points, respectively, over the negative control group with PBS‐challenge (VLP + PBS) (Figure 1B). Moreover, the total Dermatitis & Pruritus score was significantly reduced upon mIL‐5‐Qβ mice (mIL‐5‐VLP + OVA) when compared to control vaccination (VLP + OVA) (Figure 1B), whereas clinical signs were absent throughout the whole observation period for anti‐IL‐5 vaccinated mice.

### 3.2 Reduction of Allergen‐Specific IgE Levels in Allergic Mice and Horses Upon IL‐5 Vaccination

#### 3.2.1 Mice

**FIGURE 2:**
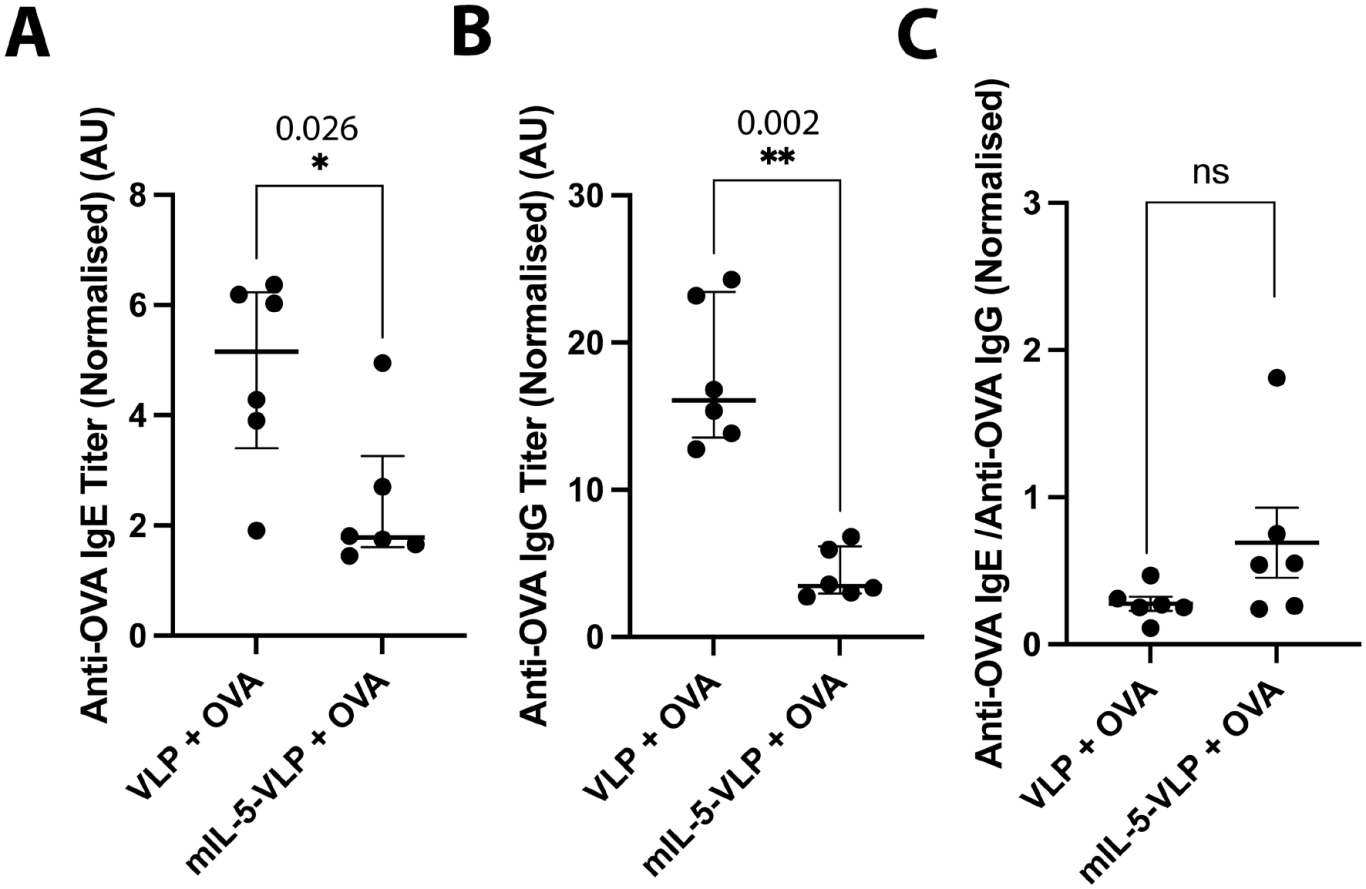
Allergen‐specific antibody responses in OVA‐sensitized and challenged mice. (A–C) Anti‐OVA IgE titer (A), anti‐OVA IgG titer (B), and anti‐OVA IgE/Anti‐OVA IgG ratio (C) at Day 22 in OVA challenged and mIL‐5‐Qβ‐immunized (mIL‐5‐VLP + OVA) and control‐CuMVTT‐vaccinated (VLP + OVA) mice and normalized to negative control with PBS challenge and CuMVTT‐vaccination (VLP + PBS). Mann–Whitney *U* test was used to compare the difference between the groups (*n* = 6 per group). Graphs are presented as median ± IQR. *p < 0.05; **p < 0.01; ***p < 0.001; ****p < 0.0001.

Besides reduction of clinical symptoms, allergen‐specific IgE and pan‐IgG were quantified in serum of mice to confirm OVA sensitization. Indeed, OVA‐challenged mice (VLP + OVA), served as positive control, showed a significant increase in anti‐OVA IgE (Figure S3A) and anti‐OVA IgG (Figure S3B), when compared to negative control group (VLP + PBS), indicating a successful OVA sensitization of OVA‐induced dermatitis mice. Anti‐mIL‐5‐vaccination using mIL‐5‐Qβ led to significantly decreased anti‐OVA IgE (Figure 2A) and IgG (Figure 2B) titer levels, when comparing mIL‐5‐vaccination to control VLP‐vaccination (data normalized to negative control group “OVA + PBS”). At the same time, no significant difference was found in the anti‐OVA IgE/anti‐OVA IgG ratio (Figure 2C).

#### 3.2.2 Horses

**FIGURE 3:**
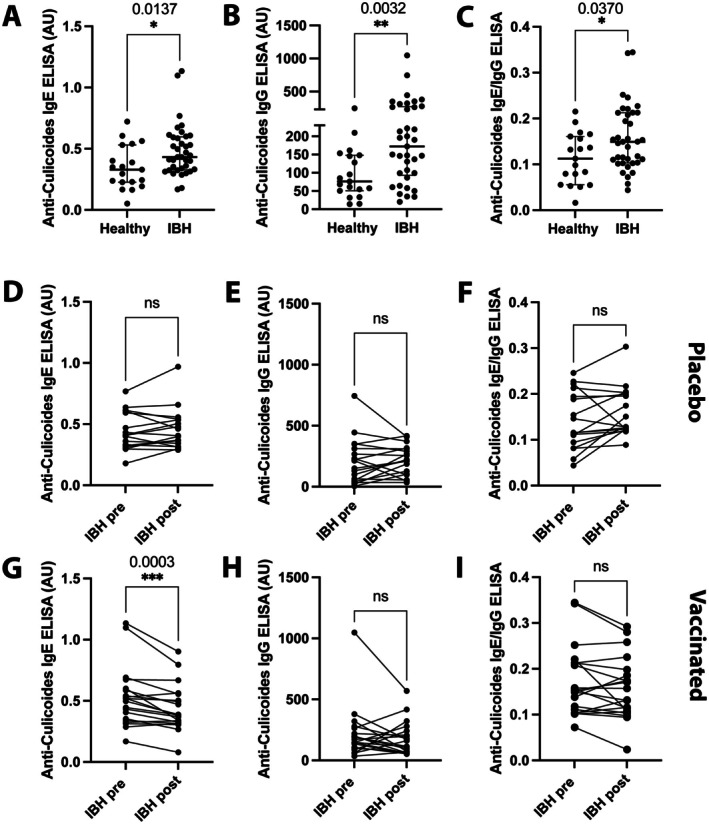
Allergen‐specific antibody responses in untreated Healthy, untreated, or placebo‐, or eIL‐5‐CuMVTT‐vaccinated IBH‐affected horses. (A–C) Anti‐*Culicoides* IgE titer (A), anti‐*Culicoides* pan‐IgG titer (B), and anti‐*Culicoides* IgE/anti‐*Culicoides* pan‐IgG ratio (C) in healthy (Healthy, *n* = 19) and untreated IBH‐affected (IBH, *n* = 37) horses. (D–F) Anti‐*Culicoides* IgE titer (D), anti‐*Culicoides* pan‐IgG titer (E), and anti‐*Culicoides* IgE/anti‐*Culicoides* pan‐IgG ratio (F) in IBH‐affected horses in untreated season (IBH pre, *n* = 17) and in subsequent placebo‐treated season (IBH post, *n* = 17). (G–I) Anti‐*Culicoides* IgE titer (G), anti‐*Culicoides* pan‐IgG titer (H), and anti‐*Culicoides* IgE/anti‐*Culicoides* pan‐IgG ratio (I) in IBH‐affected horses in untreated season (IBH pre, *n* = 20) and in subsequent eIL‐5‐CuMVTT‐vaccinated season (IBH post, *n* = 20). Mann–Whitney *U* test and Wilcoxon matched pairs test were used to compare the difference between the groups. Data are presented as median ± IQR. *p < 0.05; **p < 0.01; ***p < 0.001; ****p < 0.0001.

Similarly, allergen‐specific IgE and pan‐IgG levels were quantified in untreated IBH‐affected horses and compared to healthy control horses. Anti‐*Culicoides* IgE (Figure 3A) and IgG (Figure 3B) quantified by ELISA were significantly increased in IBH‐affected horses when compared to healthy controls. The ratio of anti‐*Culicoides* IgE to anti‐*Culicoides* IgG was significantly higher in IBH‐affected horses compared to healthy horses (Figure 3C). Further, pair analysis was conducted on eIL‐5‐CuMVTT‐vaccinated IBH horses (*n* = 20) and placebo‐treated IBH horses (*n* = 17), comparing pre‐ and post‐vaccination for placebo and vaccine horses. Our data showed no significant change in anti‐*Culicoides* IgE (Figure 3D) and IgG (Figure 3E) levels in placebo‐treated IBH horses. In addition, there was no change in the ratio of anti‐*Culicoides* IgE to pan‐IgG in the placebo group (Figure 3F).

Comparably to the mouse model, also for eIL‐5‐CuMVTT‐vaccinated IBH horses, there was a significant decrease in anti‐*Culicoides* IgE levels (Figure 3G) compared to the pre‐vaccine baseline. No significant change was observed in anti‐*Culicoides* IgG levels (Figure 3H), no change was observed in the ratio of anti‐*Culicoides* IgE to IgG in vaccinated horses when compared to baseline (Figure 3I).

**FIGURE 4:**
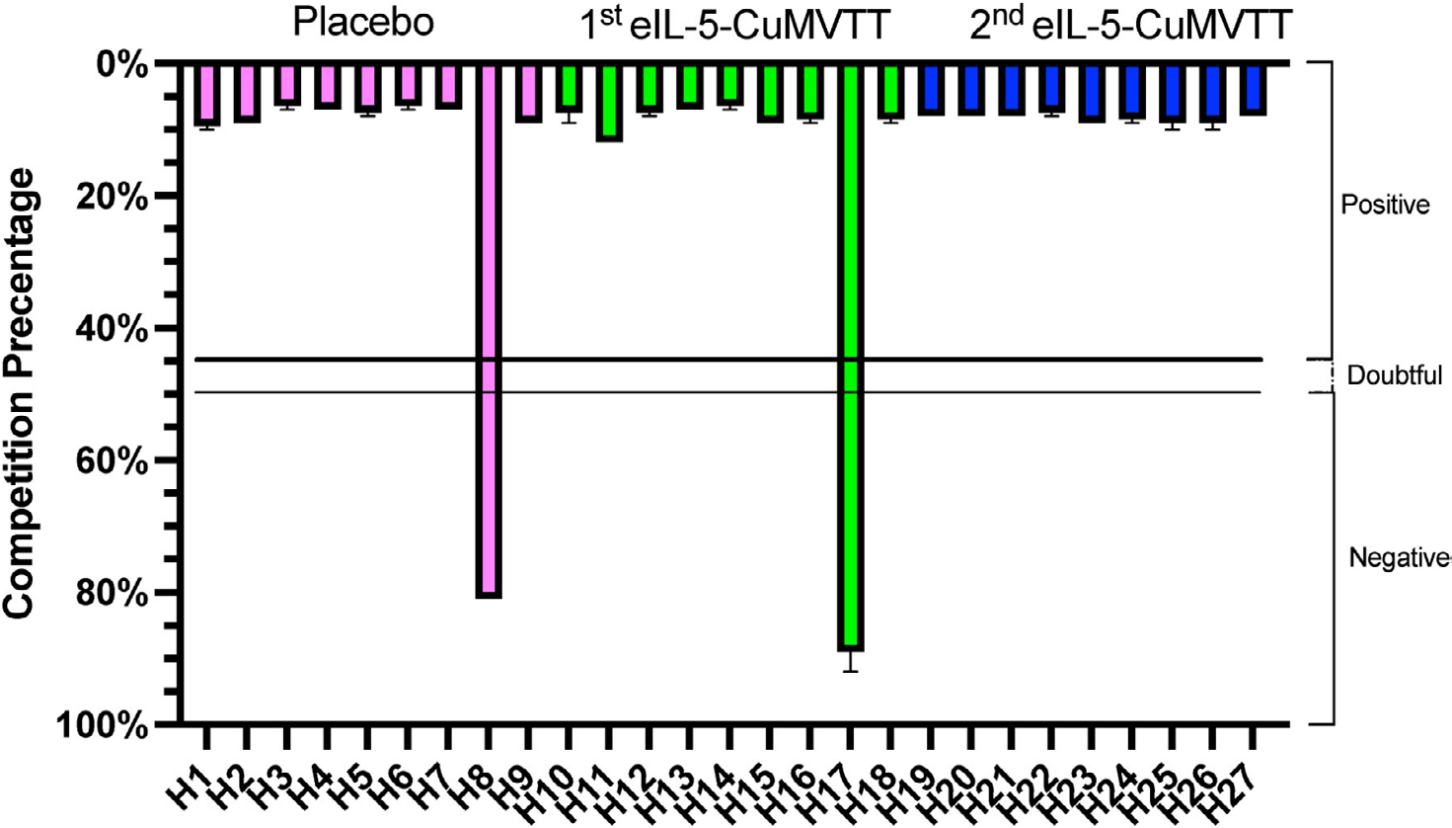
Influenza antibody responses in eIL‐5‐vaccinated horses. Influenza A nucleoprotein competition ELISA presented as competition percentage in IBH placebo‐treated horses (*n* = 9) and eIL‐5‐CuMVTT‐vaccinated horses in 1st (*n* = 9) and 2nd year (*n* = 9) of vaccination. Data are presented as median ± IQR.

To investigate whether IL‐5 vaccination affected the immune system's ability to perform class switching of antibodies, we compared levels of influenza IgG antibodies in placebo and IL‐5‐vaccinated horses. For IL‐5‐vaccinated horses, we included horses that have received an influenza vaccination after IL‐5 immunity was established; and included 1st (basic vaccination after prime‐boost schedule) and 2nd (booster) years of eIL‐5‐CuMVTT‐vaccinated horses. Our data showed that overall 92.59% of influenza‐vaccinated horses were seropositive for influenza A‐NP using a competitive influenza A‐NP ELISA setup (Figure 4). Placebo‐control and eIL‐5‐CuMVTT‐vaccinated horses in the 1st and 2nd years showed comparable competition percentages, indicating no difference in influenza A‐NP IgG due to eIL‐5‐CuMVTT vaccination. Notably, horse H8 from the placebo group and horse H17 from the eIL‐5‐CuMVTT 1st‐year vaccination group showed a negative competition A‐NP ELISA, whereas all horses in 2nd‐year vaccination showed competing influenza‐specific IgG antibodies.

### 3.3 IL‐5 Vaccination Decreased Th1/Th2 Cytokines in Allergic Mice and Horses

#### 3.3.1 Mice

**FIGURE 5:**
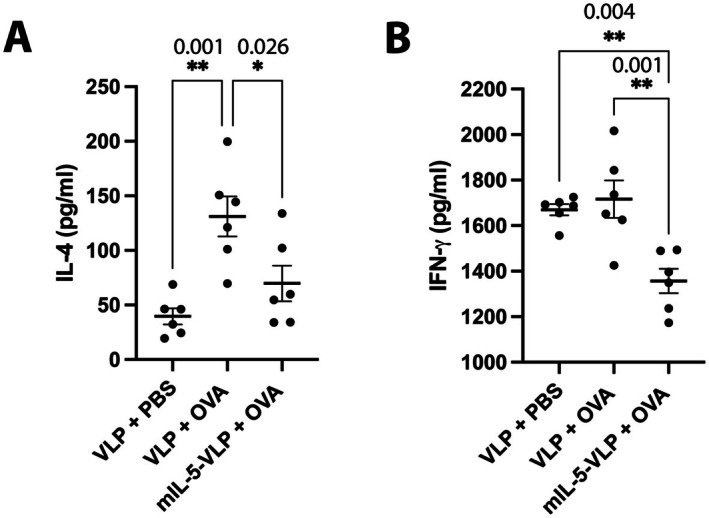
IL‐4 and IFNγ protein quantification upon splenocyte re‐stimulation with OVA in vitro from OVA‐sensitized and challenged mice. (A) IL‐4 protein quantification of in vitro re‐stimulated splenocytes of VLP‐control vaccinated mice either sensitized with OVA (VLP + OVA) or with PBS (VLP + PBS), and OVA‐sensitized mice vaccinated with mIL‐5‐Qβ (mIL‐5‐Qβ + OVA). (B) IFNγ protein quantification of in vitro re‐stimulated splenocytes of VLP‐control vaccinated mice either sensitized with OVA (VLP + OVA) or with PBS (VLP + PBS), and OVA‐sensitized mice vaccinated with mIL‐5‐Qβ (mIL‐5‐Qβ + OVA). Ordinary one‐way ANOVA with Tukey's multiple comparisons test was used to compare the difference between the groups (*n* = 6 per group). Data are presented as mean ± SEM. *p < 0.05; **p < 0.01; ***p < 0.001; ****p < 0.0001.

Spleens from all mouse groups were collected, splenocytes were isolated, and OVA re‐stimulated in vitro. A significant increase in IL‐4 protein expression levels was found for OVA‐challenged mice (VLP + OVA) when compared to the negative PBS‐challenge group (VLP + PBS) (Figure 5A). In contrast, for the OVA‐challenged mice, the mIL‐5‐Qβ‐vaccinated mice showed a significant decrease in IL‐4 levels when compared to the VLP‐control‐vaccinated mice (Figure 5A). In addition, IFNγ protein expression did not change upon OVA‐challenge, comparing VLP + OVA mice to the PBS‐challenged negative group VLP + PBS (Figure 5B). However, comparing the two OVA‐challenged mouse groups, the mIL‐5‐Qβ‐vaccinated mice showed a significant decrease in IFNγ levels when compared to the VLP‐vaccinated control group (Figure 5B).

#### 3.3.2 Horses

**FIGURE 6:**
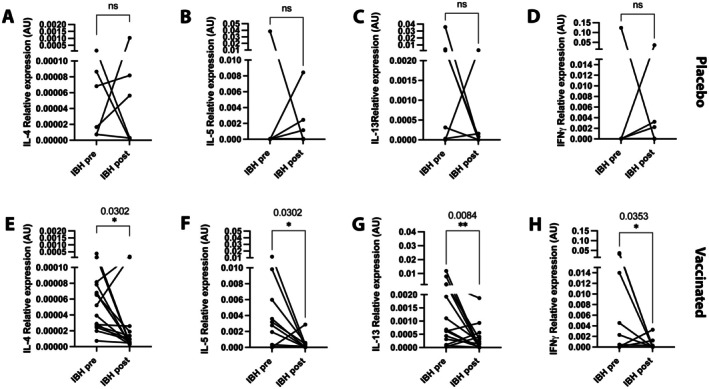
Th1 and Th2 cytokine mRNA levels decrease in skin punch biopsies of IBH horses upon eIL‐5‐CuMVTT‐vaccination. (A–D) IBH‐affected horses in untreated season (IBH pre‐placebo, *n* = 6) and in subsequent placebo‐treated season (IBH post‐placebo, *n* = 6) with individual expression levels for mRNA ($2^{-∆C_{t}}$) of IL‐4 (A), IL‐5 (B), IL‐13 (C), and IFNγ (D). (E–H) IBH‐affected horses in untreated season (IBH pre‐vaccination, *n* = 15) and in subsequent eIL‐5‐CuMVTT‐vaccinated season (IBH post‐vaccination, *n* = 15) with individual expression levels for mRNA ($2^{-∆C_{t}}$) of IL‐4 (E), IL‐5 (F), IL‐13 (G), and IFNγ (H). Delta *C*_t_ was calculated using *C*_t_ gene of interest—*C*_t_ GAPDH (GAPDH variability was low comparing to other endogenous control genes and thus was chosen as house‐keeping control). Wilcoxon matched pairs test was used to compare the difference between the groups. *p < 0.05; **p < 0.01; ***p < 0.001; ****p < 0.0001.

Th1/Th2 mRNA cytokine expression was quantified in lesional skin punch biopsies from IBH‐affected horses before and after vaccination in the placebo‐treated group. Our data showed no significant change in IL‐4 (Figure 6A), IL‐5 (Figure 6B), IL‐13 (Figure 6C) or IFNγ (Figure 6D) mRNA expression in the skin of IBH‐affected horses before and after placebo treatment. However, in eIL‐5‐CuMVTT‐vaccinated IBH horses, there was a significant decrease in IL‐4 (Figure 6E), IL‐5 (Figure 6F), IL‐13 (Figure 6G), and IFNγ (Figure 6H) mRNA expression levels when compared to the skin of the same horses prior to vaccination.

### 3.4 Th2 Cells Are the Main Producers of IL‐5 and CD4 ^−^ MHCII ^−^ Cells of IL‐4 Upon Allergen Stimulation In Vitro in IBH Horses

**FIGURE 7:**
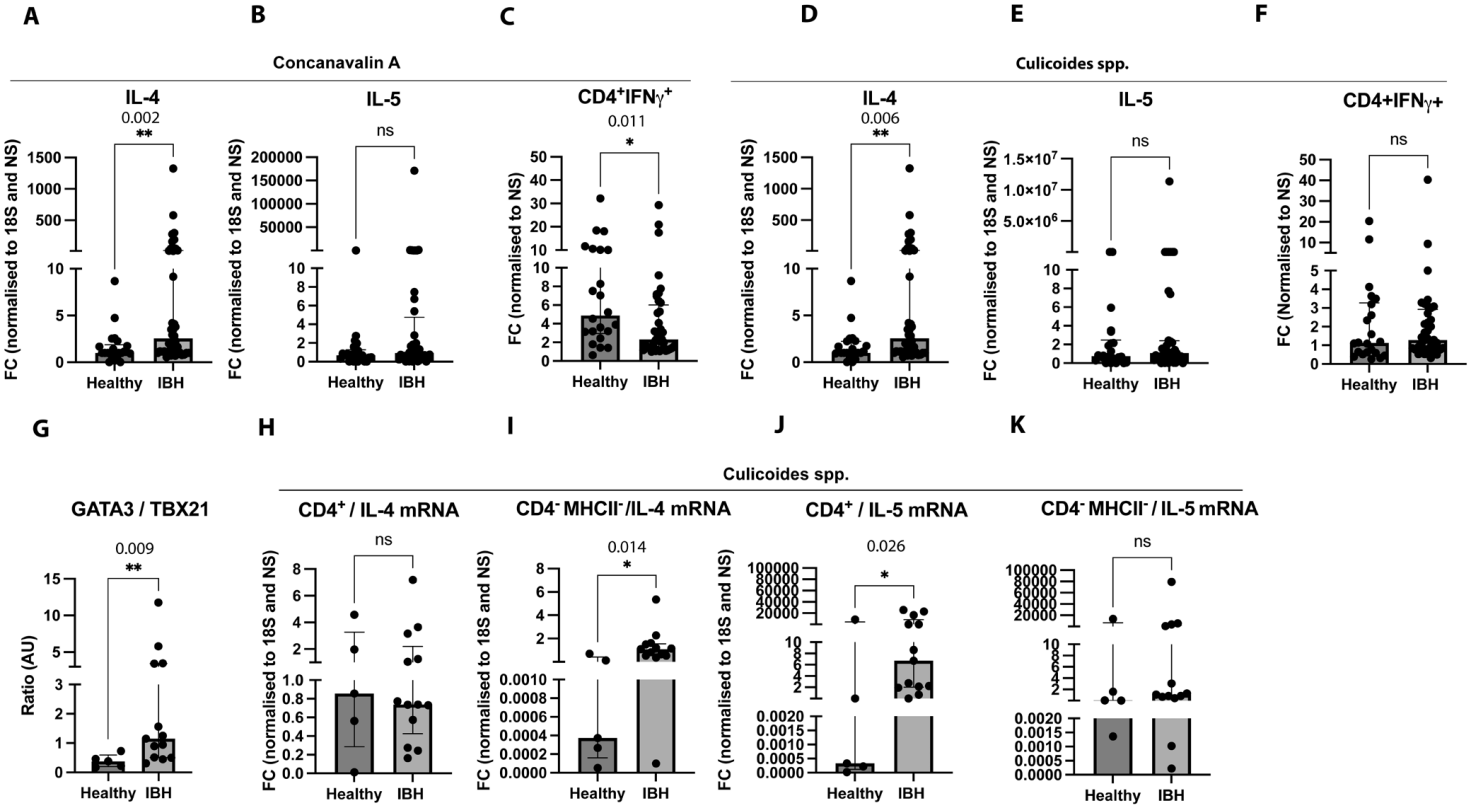
Th1/2 cell subpopulation and cytokines in CD4^+^MHC^−^ and CD4^−^MHC^−^ sorted cells upon in vitro stimulation with ConA and *Culicoides* in untreated healthy and untreated IBH horses. For mRNA, data normalized to endogen control 18S and non‐stimulated condition (IL‐4, IL‐5); for protein, data normalized to non‐stimulated (IFNγ). (A–C) IL‐4 (A), IL‐5 (B) mRNA fold change and IFNγ protein expression (C) in IBH (*n* = 40) and healthy (*n* = 22) PBMCs stimulated with ConA (1 μg/mL). (D–F) IL‐4 (D), IL‐5 (E) mRNA fold change and IFNγ protein expression (F) in IBH (*n* = 40) and healthy (*n* = 22) PBMCs stimulated with *Culicoides* (1 μg/mL). (G) GATA3/TBX21 mRNA expression ratio in IBH (*n* = 13) and healthy (*n* = 5) sorted CD4^+^ cells in non‐stimulated condition. (H–K) IL‐4 and IL‐5 mRNA expression in sorted CD4^+^ cells and sorted CD4^−^MHCII^−^. IL‐4 mRNA expression in sorted CD4^+^ cells (H) and CD4^−^MHCII^−^ (I) from IBH (*n* = 13) and healthy (*n* = 5) horses after stimulation with *Culicoides* (1 μg/mL). IL‐5 mRNA expression in sorted CD4^+^ cells (J) and CD4^−^MHCII^−^ (K) from IBH (*n* = 13) and healthy (*n* = 5) horses after stimulation with *Culicoides* (1 μg/mL). Mann–Whitney *U* test was used to compare the difference between the groups. Data are presented as median ± IQR. *p < 0.05; **p < 0.01; ***p < 0.001; ****p < 0.0001.

PBMCs from the blood of untreated IBH‐affected and healthy horses were stimulated in vitro with ConA (1 μg/mL) or *Culicoides* (1 μg/mL) to assess their response to antigen‐independent or allergen‐dependent stimulation, respectively. Furthermore, ConA or *Culicoides* stimulated cells were then either used for (i) RNA isolation of total PBMCs and analyzed for IL‐4 and IL‐5 mRNA expression by qPCR, (ii) intracellular IFNγ expression of CD4^+^ cells by flow cytometry, or (iii) sorting of CD4^+^ and CD4^−^ cells with subsequent RNA isolation and analyzed for IL‐4 and IL‐5 mRNA expression by qPCR, aiming to identify the source of IL‐4 and IL‐5 mRNA cytokine production. PBMC stimulation with ConA and subsequent total PBMC RNA extraction showed a significant increase in IL‐4 mRNA expression in IBH horses compared to healthy horses (Figure 7A) and no significant change was observed in IL‐5 mRNA expression (Figure 7B). Intracellular IFNγ of CD4^+^ cells, however, was significantly decreased in IBH‐affected horses compared to healthy horses (Figure 7C). When PBMCs were re‐stimulated with *Culicoides* instead, there was a significant increase in IL‐4 mRNA expression in IBH horses compared to healthy horses, comparable to what was found for the antigen‐independent ConA stimulation (Figure 7D). Also, no significant change in IL‐5 mRNA expression was found for *Culicoides* stimulation of healthy and IBH horses (Figure 7E). In contrast to the ConA stimulation, IFNγ protein expression was comparable for healthy and IBH horses upon *Culicoides* stimulation (Figure 7F).

To assess the Th2/Th1 dominance in IBH‐affected and healthy horses and investigate the allergen response of CD4^+^ and CD4^−^MHCII^−^ cells in terms of IL‐4 and IL‐5 mRNA production, PBMCs were stained and sorted after stimulation with *Culicoides* at 1 μg/mL, using a gating strategy as shown in Figure S3. Unstimulated CD4^+^ cells from IBH‐affected horses showed a significant increase in GATA3/TBX21 ratio (Figure 7G) when compared to healthy horses, primarily due to the downregulation of TBX21 (Figure S4A) while GATA3 expression remained unchanged (Figure S4B). Upon stimulation with *Culicoides*, CD4^+^ cells showed no significant change in IL‐4 mRNA expression comparing healthy and IBH horses (Figure 7H), whereas sorted CD4^−^MHCII^−^ cells showed a significant increase in IL‐4 mRNA expression in IBH‐affected horses when compared to healthy horses (Figure 7I). Furthermore, sorted CD4^+^ cells showed a significant upregulation of IL‐5 mRNA expression in IBH‐affected horses compared to healthy horses (Figure 7J), whereas no significant change was observed in IL‐5 mRNA expression in sorted CD4^−^MHCII^−^ cells (Figure 7K).

## 4 Discussion

In previous studies, we demonstrated that eIL‐5‐CuMVTT vaccination in IBH‐affected horses successfully depleted blood eosinophilia [19, 20, 35], particularly the inflammatory eosinophil (iEos) subset [35], leading to a significant reduction in IBH lesion scores [19, 20]. Furthermore, eIL‐5‐CuMVTT vaccination significantly decreased basophil counts in IBH‐vaccinated horses over a 2‐year treatment period [22]. In the present study, we investigated the effects of IL‐5 vaccination on allergen‐specific IgE levels and Th1/Th2 cytokine modulation in two species with skin allergy: an induced skin allergy model in mice using ovalbumin mimicking allergic dermatitis; and a naturally occurring equine skin allergy, IBH, an allergy towards insect bites. Our data showed that vaccination with IL‐5 significantly decreased allergen‐specific IgE levels and modulated Th1/Th2 cytokine profiles. Furthermore, in allergic horses we identified Th2 cells as the main source of IL‐5, whereas CD4^−^MHCII^−^ cells acted as the main source of IL‐4.

Intriguingly, allergic animals vaccinated with IL‐5 showed a significant drop in allergen‐specific IgE, along with an alleviation of clinical signs when compared to the control group. Recently, Nunomura et al. suggested a new mechanism by which benralizumab, targeting the human (hu) IL‐5 receptor alpha (IL‐5Ra), reduced allergen‐specific IgE levels. This is achieved by decreasing IL‐4 expression in human mast cells in peripheral blood and IL‐13 in a specific mast cell (MC3) subpopulation in the lungs [36]. Benralizumab showed a significant reduction in allergen‐specific IgE levels post‐treatment (116 ± 3 days, −35%), whereas the anti‐huIL‐5 monoclonal antibody, mepolizumab, did not affect IgE levels in the blood [37]. Of note, and yet to our knowledge, there is no data on IgE available for reslizumab, the other authorized anti‐huIL‐5 monoclonal antibody. Further, the authors claimed that both treatment groups showed clinical improvements in their asthma control test (ACT) scores and observed a significant reduction in eosinophils, with no effect on lymphocyte and neutrophil counts. Moreover, they noted a more substantial reduction in basophils with benralizumab (−78%) compared to mepolizumab (−33%) [37]. Their data demonstrated that targeting the IL‐5 receptor using benralizumab reduced the percentages of B cells expressing IGHE germline transcripts in peripheral blood and lungs. Indeed, IL‐5Ra was found to be expressed in a subset of B cells [38, 39], also granulocytes (eosinophils, basophils and neutrophils [40, 41, 42]), and mast cells [43, 44]. Further, Kolbeck and Contoli showed that benralizumab depleted eosinophils and basophils in non‐human primates and human patients with eosinophilic asthma through enhanced antibody‐dependent cell‐mediated cytotoxicity (ADCC) [37, 40, 45, 46]. In our study, the VLP‐based IL‐5 vaccination showed a similar effect as benralizumab, with significantly decreasing allergen‐specific serum IgE levels and in the 2nd year of vaccination only a significant reduction of basophil cell counts in blood [22]. Add to that, we also showed a phenotypic shift of inflammatory eosinophil subset towards a resident healthy‐like phenotype after 2 years of eIL‐5‐CuMVTT vaccination [21].

Besides the reduction of allergen‐specific IgE levels upon IL‐5 vaccination in allergic mice and horses, alterations in T cell cytokine expression were found also for both species. OVA‐sensitized mice vaccinated with mIL‐5‐VLP showed a significant decrease in IL‐4 and IFNγ protein expression in splenocytes re‐stimulated with OVA. In horse skin biopsies, eIL‐5‐vaccinated IBH horses revealed a significant decrease of IL‐4, IL‐5, IL‐13, and IFNγ mRNA when compared to the skin of the same horses prior to vaccination. Along the same line, we demonstrated in an earlier publication that skin punch biopsies from IBH‐affected horses showed significantly elevated levels of Th1/Th2 cytokines, including IL‐4, IL‐5, IL‐13, and IFNγ mRNA, compared to healthy horses [47].

Across both murine and equine allergy, our data consistently showed an increase in allergen‐specific IgE levels in allergic individuals, which was accompanied by elevated IL‐4 levels, found in equine skin biopsies [47], mouse splenocytes, and equine PBMCs upon re‐stimulation with OVA or *Culicoides*, respectively. Furthermore, *Culicoides‐*stimulated PBMCs sorted for CD4^+^ and CD4^−^ cells from IBH and healthy horses showed that CD4^−^MHCII^−^ cells are the primary producers of IL‐4, whereas CD4^+^ cells produced a significant amount of IL‐5. These IL‐5‐producing CD4^+^ cells may potentially represent peTh2 cells, which are likely to be responsible for the key involvement of eosinophils in the pathology of IBH [19]. Regarding the rather surprising finding of non‐Th2 cells but CD4^−^MHCII^−^ cells being the primary source for IL‐4, this finding is aligned with the findings of a previous study [48]; the source and regulation of IL‐4, as well as its responsiveness towards the IL‐5 vaccination, are essential for a comprehensive understanding of the pathology. IL‐4 is a pleiotropic cytokine produced by various cell types including Th2 cells [49, 50], natural killer T cells [51, 52], innate immune cells type‐2 [53, 54], mast cells [55, 56], eosinophils [57, 58, 59] and basophils [60, 61, 62]. Notably, mast cells circulate in the blood as immature progenitors. However, in the allergy letter by Nunomura et al., the authors demonstrated the presence of mast cells in the peripheral blood of humanized mice, particularly among hCD45^+^ cells, using scRNA‐seq technology. Additionally, they found three distinct mast cell subsets in hCD45^+^ lung cells that were absent in hCD45^+^ peripheral blood cells. Furthermore, the mast cell population in the peripheral blood was shown to express IL‐4, which was significantly decreased in benralizumab‐treated mice compared to hIgG1 control mice. This could be explained by the fact that circulating mast cells expressing IL‐5Ra and IL‐4 may be undergoing a transitional differentiation phase in blood, induced by inflammatory stimuli, in preparation for migration to the tissue [36]. Recent research supports that blood eosinophils in mice also do not express CD4 or MHCII [34], whereas basophils lack CD4 [63, 64] their MHCII expression remains controversial [65, 66, 67, 68]. Given these findings, CD4^−^MHCII^−^ cells producing IL‐4 in our study could be eosinophils, basophils, ILC‐2, or mast cells. Interestingly, another study suggests that basophils are the primary source of early IL‐4 secretion following *Culicoides* allergen stimulation in vitro [48]. With regard to the IL‐5 vaccine‐induced IL‐4 dampening, the question remains how this is regulated and whether the effect is a direct effect of the IL‐5 removal or caused indirectly by the reduction of eosinophils and potentially ILC‐2 cells in the blood and tissue. Adding the reduction of basophils in blood found in the second IL‐5 vaccination year, the IL‐4 reduction might be linked to this finding.

Interestingly, a significant decrease in IFNγ protein expression was observed upon stimulation with ConA, not *Culicoides*, in PBMCs of IBH compared to healthy horses. These results could be explained by the Th2 enrichment in allergic horses, as reflected by a higher GATA3/TBX21 ratio in sorted CD4^+^ cells, due to the decrease of TBX21 in IBH compared to healthy horses. The findings suggest that IL‐5 plays a central role in the allergic response, particularly in altering levels of IL‐4 and directly or indirectly driving the production of allergen‐specific IgE, whereas IFNγ appears to be less involved in this process. Taking together, the IL‐5 vaccination may cause the following turn of events: neutralizing anti‐IL‐5 antibodies decrease T‐cell‐derived levels of IL‐5, which shuts down the de novo eosinophil production; the reduced levels of IL‐5 or the reduced availability of eosinophils in the tissue may then lead to lower eosinophil‐derived IL‐4 levels or lower IL‐4 levels derived from cells responsive to IL‐5 or lacking feedback from eosinophils; the lower IL‐4 levels may impact allergen‐specific IgE levels. Furthermore, all players in allergy interact on various levels, and shutting down one cell type will eventually have an effect on the other cells by receiving fewer signals (indirect effect). In addition, other IL‐5R‐expressing cells may also be affected by lower abundant IL‐5 levels (direct effect), maybe causing them to produce lower levels of downstream molecules.

Previous studies have established that IgE synthesis is regulated by several factors including cytokines. Among these, IL‐4 is particularly well known for inducing IgE secretion [69, 70, 71, 72, 73] by promoting the switch from IgM to IgE [74] and IgG1 [75] in resting B cells. Similarly, IL‐5 contributes to promoting B cell differentiation and immunoglobulin (Ig) switching [75, 76, 77].

It is generally known that Ig class switching by B cells can be T cell dependent [78, 79] or independent (by the induction of lipopolysaccharide) [80, 81, 82]. Furthermore, Purkerson and Isakson [83] showed that IL‐5 provides a signal that is required in addition to the IL‐4 signal for isotype switching to IgG1 and IgE. Indeed, our data for the mouse model aligned with Purkerson's findings while IL‐5‐vaccinated OVA‐sensitized mice showed decreased OVA‐specific IgE and IgG levels. By contrast, the therapeutic IL‐5 vaccination of naturally allergic horses did only reveal a significant reduction for allergen‐specific IgE levels, and not for the IgG. The OVA allergic mouse model is an experimental induction of a Th2‐skewed immune response triggering a strong antibody production. This may be the reason for differing allergen‐specific IgG results in mice and horses. To control for B cells being not generally impaired in their ability to perform Ig class switching caused by an IL‐5‐vaccine‐induced lack of IL‐5 and a consequently interlinked lack of IL‐4, we investigated the ability of IL‐5‐vaccinated horses to respond to a seasonal influenza vaccine. Induction of influenza‐specific IgG antibodies was compared for IL‐5‐ and placebo‐vaccinated horses and showed comparable results, even in horses being IL‐5 vaccinated in two subsequent years. Consistent with a previous study, Jonsdottir et al. [23] showed that tetanus‐specific IgG levels, in isolated PBMCs stimulated with resquimod, a toll‐like receptor‐7 agonist, did not differ between eIL‐5 vaccinated and unvaccinated IBH horses. Thus, the decrease of allergen‐specific IgE levels upon IL‐5 vaccination while allergen‐specific IgG remained unchanged indicates further that there is no inhibition of B cell class switching upon IL‐5 vaccination.

On the role of IL‐5 on allergen‐specific IgE levels, the question of whether to use the IL‐5 vaccine not only therapeutically but also prophylactically may arise. Along these lines, Kung et al. [84] showed a prophylactic and therapeutic effect of an anti‐IL‐5 antibody (TRFK‐5) in a murine model of allergic pulmonary inflammation. They found a dose‐dependent inhibition of the pulmonary eosinophilia by TRFK‐5 when administrated prior to antigen challenge. Most importantly, they did observe an even greater inhibition on BAL eosinophils together with a reduction of the lung tissue damage severity when TRFK‐5 was administrated prior to the allergen challenge. Thus, an IL‐5 vaccination could be used prophylactically on high‐risk horses, such as Icelandic horses exported to *Culicoides*‐infested areas, as those horses lack immunological tolerance due to absence of *Culicoides* midges during early days of life and are high‐risk candidates to develop IBH [85, 86, 87, 88, 89].

In conclusion, using mice and ovalbumin mimicking allergic dermatitis and using IBH, a natural equine skin allergic disease, we demonstrated that IL‐5 vaccination significantly decreased allergen‐specific IgE levels, together with Th1/Th2 cytokines, such as IL‐4 and IFNγ. In allergic animals, we could demonstrate that innate immune cells are the main source of IL‐4 and Th2 cells are the main source of IL‐5, respectively. Thus, targeting IL‐5 with vaccination proves to be both effective and safe over the long term and shows a big potential to be used for a prophylactic effect on exported horses to *Culicoides*‐infested areas. Further research is required to investigate the influence of anti‐IL‐5 vaccination‐induced reduction of allergen‐specific IgE, IL‐4, and IL‐5 on a functional level by the degranulation of effector cells.

## Author Contributions

Conceptualization, A.F.‐G.; methodology, F.J., F.O., V.I., G.K., T.R., N.W., J.L., S.P., F.C., and A.F.‐G.; software, F.J. and A.F.‐G.; validation, F.J. and A.F.‐G.; formal analysis, F.J. and A.F.‐G.; investigation, F.J. and A.F.‐G.; resources, T.M.K. and A.F.‐G.; data curation, F.J. and A.F.‐G.; writing – original draft preparation, F.J. and A.F.‐G.; writing – review and editing, F.J., F.O., V.I., G.K., T.R., N.W., J.L., S.P., F.C., K.B., P.J., T.M.K., and A.F.‐G.; visualization, F.J. and A.F.‐G.; supervision, A.F.‐G.; project administration, A.F.‐G.; funding acquisition, A.F.‐G. All authors have read and agreed to the published version of the manuscript.

## Conflicts of Interest

F.J., V.I., G.K., T.R., N.W., J.L., F.C., K.B., T.M.K., and A.F.‐G. are involved in the development of therapeutic equine vaccines. F.O., S.P., and P.J. have no financial or personal conflicts of interest.

## Supporting information


**Figure S1:** all70020‐sup‐0001‐FiguresS1‐S4.zip.
**Figure S2:** all70020‐sup‐0001‐FiguresS1‐S4.zip.
**Figure S3:** all70020‐sup‐0001‐FiguresS1‐S4.zip.
**Figure S4:** all70020‐sup‐0001‐FiguresS1‐S4.zip.

**Table S1:** all70020‐sup‐0002‐TableS1.xlsx.

## Data Availability

The data that support the findings of this study are available from the corresponding author upon reasonable request.
