# Supplementary material for: Anti‐IL‐5 Vaccination Dampens Allergen‐Specific IgE Levels and Modulates IL‐4 and IL‐5 Th2 Cytokines in Skin Allergy of Mice and Horses
Source: Allergy. 2025 Aug 21;80(12):3377–90. doi: 10.1111/all.70020 (PMC12666748; doi:10.1111/all.70020)
Supplement: Supplementary file 1 — Figure S1: all70020‐sup‐0001‐FiguresS1‐S4.zip. Figure S2: all70020‐sup‐0001‐FiguresS1‐S4.zip. Figure S3: all70020‐sup‐0001‐FiguresS1‐S4.zip. Figure S4: all70020‐sup‐0001‐FiguresS1‐S4.zip. [file ALL-80-3377-s001.zip › all70020-sup-0001-AppendixS1.docx]

# **Supplementary Information**

**Anti-IL-5 vaccination dampens allergen-specific IgE levels and modulates IL-4 and IL-5 Th2 cytokine in skin allergy of mice and horses**

# **Figure Legends**

**Supplementary Figure 1:** Experimental scheme and timeline of mIL-5-Qβ or control-CuMVTT-VLP vaccination and the MC903-AD-mouse model with OVA-sensitization by i.p. injection of OVA/alum and topical sensitization on the right ear, topical OVA- or PBS-challenge on the left ear including the ear thickness measurement and Dermatitis & Pruritus scoring, blood and organ collection of the three groups (n=6 mice per group); group 1, control-VLP vaccination and PBS challenge (VLP + PBS), group 2, control-VLP vaccination and OVA challenge (VLP + OVA), mIL-5-VLP vaccination and OVA challenge (mIL-5-VLP + OVA).

**Supplementary Figure 2:** Representative of gating/sorting strategy on CD4^+^ and CD4^-^ MHCII^-^ cells. **A.** Lymphocytes gated based on SSC-A and FSC-A, followed by exclusion of double cells by gating on SSC-H and FSC-H then MHC-II^+^ cells were excluded as antigen presenting and B cells. Then CD4 MHC-II^-^ cells were divided into CD4^+^MHC-II^-^ and CD4^-^MHC-II^-^ cells. **B.** Post-sorting purity shown in untreated healthy and untreated IBH horses, where purity assessment of sorted cells represented as percentage of live single lymphocytes.

**Supplementary Figure 3: Allergen-specific antibody responses in mice and influenza antibody responses in eIL-5-vaccinated horses. (A. & B.) Mice.** Anti-OVA IgE titer (**A**) and anti-OVA IgG titer (**B**) at day 22 in PBS (VLP + PBS) or OVA (VLP + OVA) challenged control CuMVTT-vaccinated mice. Graphs are presented as median ± IQR. *P<0.05; **P<0.01; ***P<0.001; ****P<0.0001.

**Supplementary Figure 4: (A. & B.)** TBX21 (**A**) and GATA3 (**B**) mRNA expression presented as 2^^-ΔCt^ in unstimulated CD4+MHC-II- cells of untreated healthy (n=5) and untreated IBH (n=13) horses. Mann-Whitney U test was used to compare the difference between the groups. Graphs are presented as median ± IQR. *P<0.05; **P<0.01; ***P<0.001; ****P<0.0001.
