# Supplementary figures and images for: Anti‐IL‐5 Vaccination Dampens Allergen‐Specific IgE Levels and Modulates IL‐4 and IL‐5 Th2 Cytokines in Skin Allergy of Mice and Horses
Source: Allergy. 2025 Aug 21;80(12):3377–90. doi: 10.1111/all.70020 (PMC12666748; doi:10.1111/all.70020)

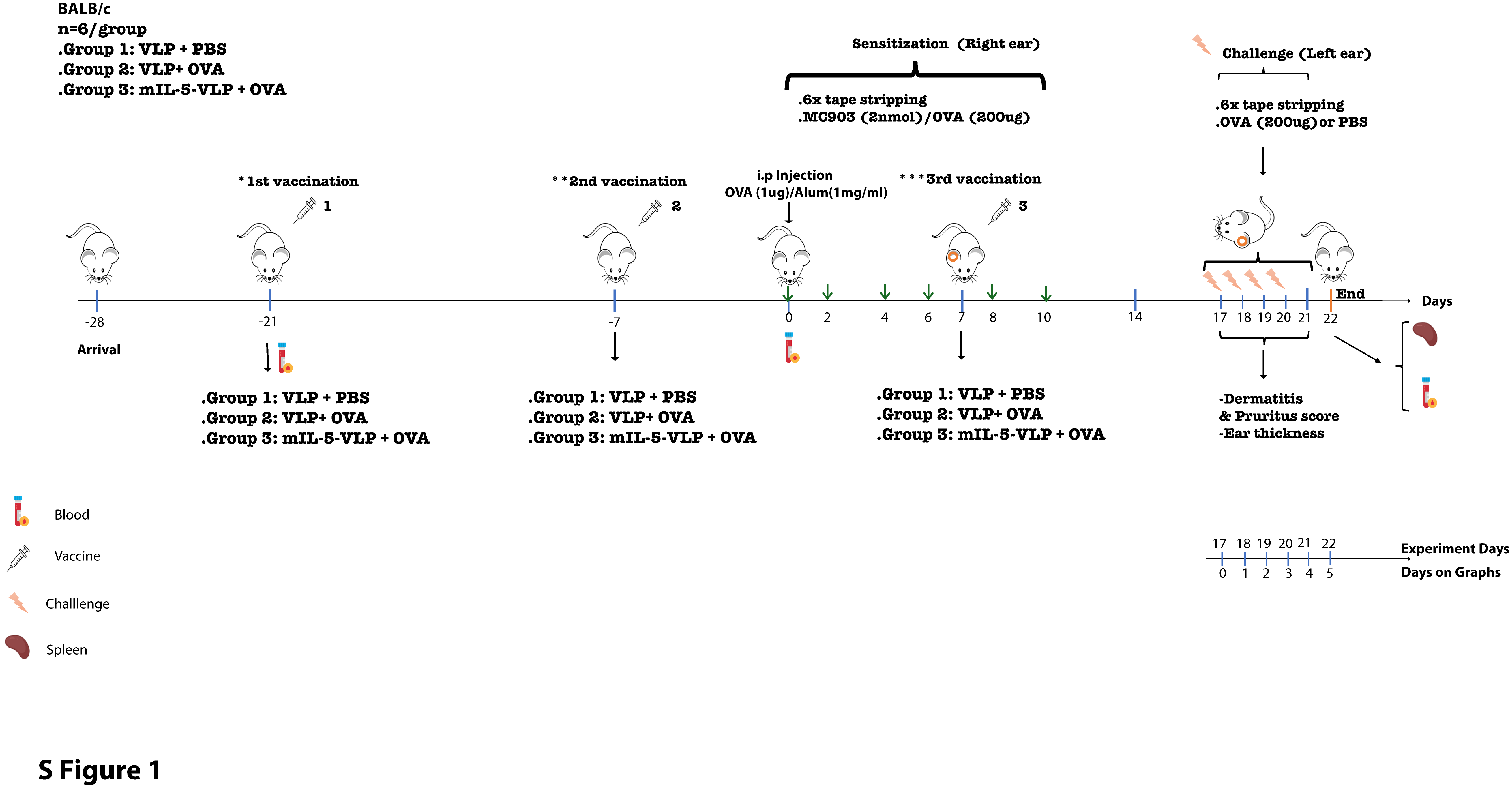

Supplement: Supplementary file 1 — Figure S1: all70020‐sup‐0001‐FiguresS1‐S4.zip. Figure S2: all70020‐sup‐0001‐FiguresS1‐S4.zip. Figure S3: all70020‐sup‐0001‐FiguresS1‐S4.zip. Figure S4: all70020‐sup‐0001‐FiguresS1‐S4.zip. [file ALL-80-3377-s001.zip › all70020-sup-0003-Supinfo1@S Figure 1.tif]

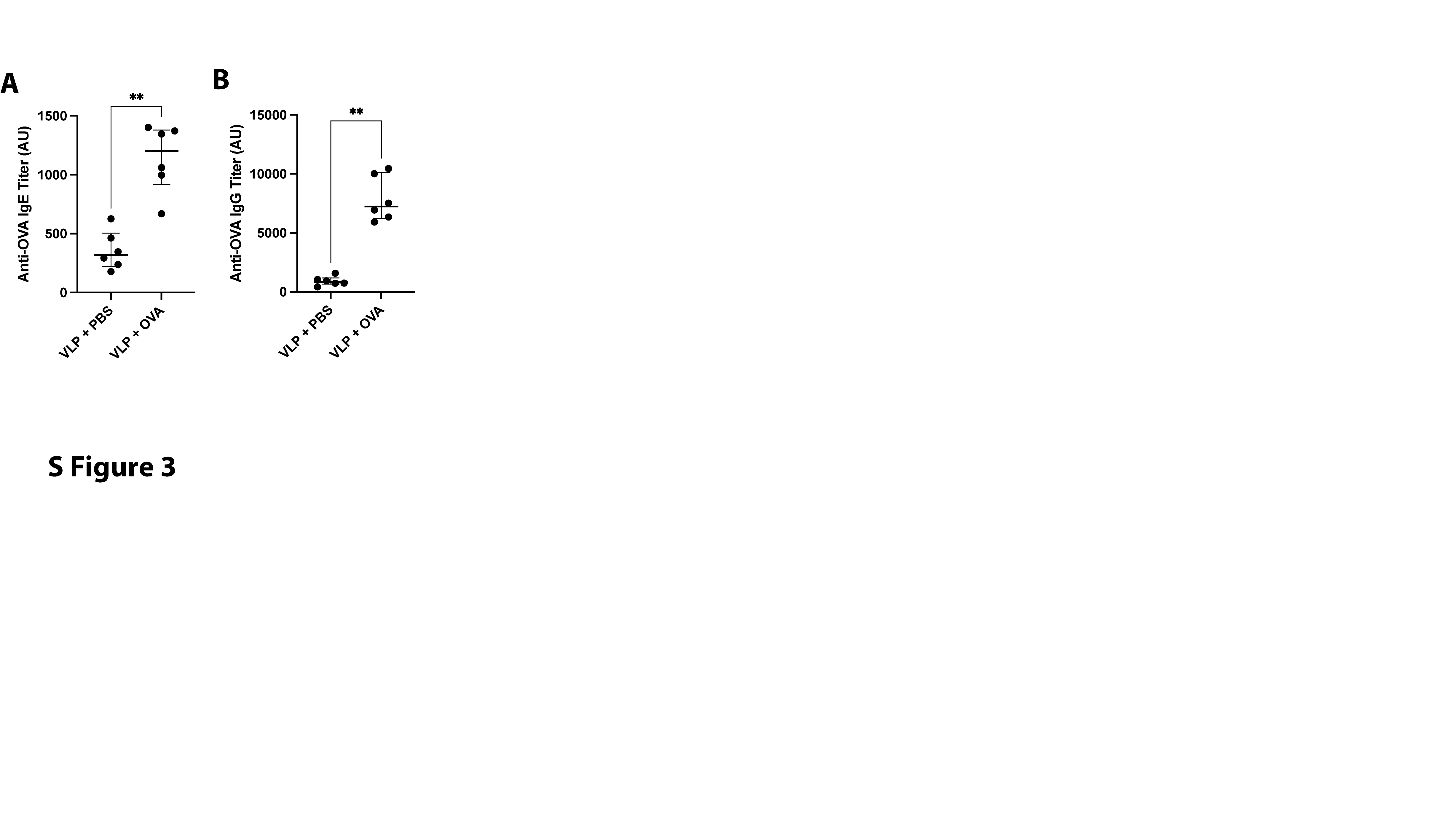

Supplement: Supplementary file 1 — Figure S1: all70020‐sup‐0001‐FiguresS1‐S4.zip. Figure S2: all70020‐sup‐0001‐FiguresS1‐S4.zip. Figure S3: all70020‐sup‐0001‐FiguresS1‐S4.zip. Figure S4: all70020‐sup‐0001‐FiguresS1‐S4.zip. [file ALL-80-3377-s001.zip › all70020-sup-0005-Supinfo3@S Figure 3.tif]

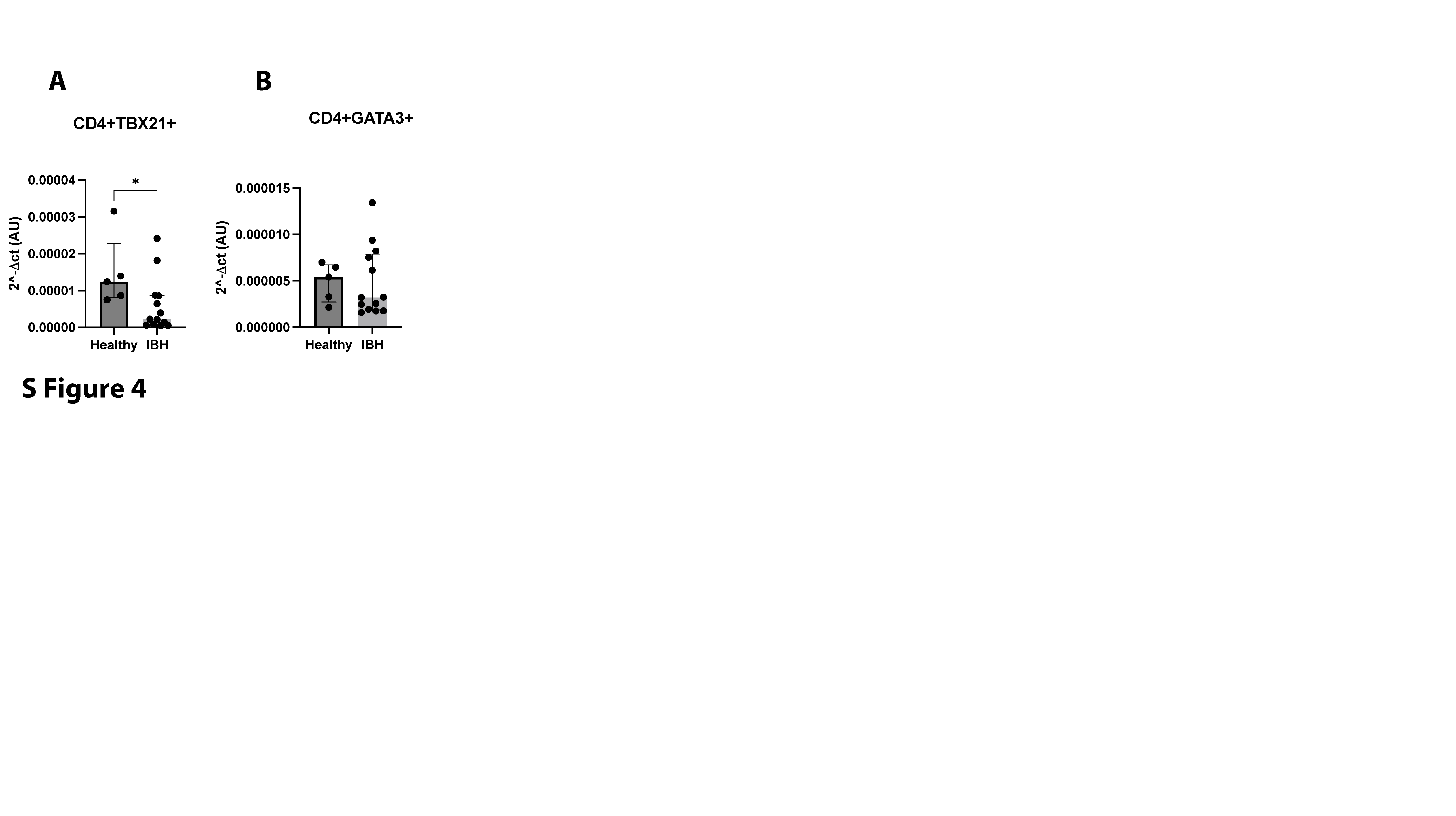

Supplement: Supplementary file 1 — Figure S1: all70020‐sup‐0001‐FiguresS1‐S4.zip. Figure S2: all70020‐sup‐0001‐FiguresS1‐S4.zip. Figure S3: all70020‐sup‐0001‐FiguresS1‐S4.zip. Figure S4: all70020‐sup‐0001‐FiguresS1‐S4.zip. [file ALL-80-3377-s001.zip › all70020-sup-0006-Supinfo4@S Figure 4.tif]
